# Relationship between gut microbiota and the pathogenesis of gestational diabetes mellitus: a systematic review

**DOI:** 10.3389/fcimb.2024.1364545

**Published:** 2024-05-28

**Authors:** Sheng Ma, Yuping Wang, Xiaoxia Ji, Sunjuan Dong, Shengnan Wang, Shuo Zhang, Feiying Deng, Jingxian Chen, Benwei Lin, Barkat Ali Khan, Weiting Liu, Kaijian Hou

**Affiliations:** ^1^Anhui Province Maternity & Child Health Hospital, Hefei, Anhui, China; ^2^School of Nursing, Anhui University of Chinese Medicine, Hefei, Anhui, China; ^3^Nursing Department, Shantou Central Hospital, Shantou, Guangdong, China; ^4^Shantou University Medical College, Shantou, Guangdong, China; ^5^School of Physiology, Pharmacology and Neuroscience, University of Bristol, Bristol, United Kingdom; ^6^Drug Delivery and Cosmetic Lab (DDCL), Gomal Center of Pharmaceutical Sciences, Faculty of Pharmacy, Gomal University, Dera Ismail Khan, Pakistan; ^7^School of Public Health, Shantou University, Shantou, Guangdong, China

**Keywords:** gestational diabetes mellitus, gut microbiota, chronic inflammatory state, insulin resistance, pathogenesis

## Abstract

**Introduction:**

Gestational diabetes mellitus (GDM) is a form of gestational diabetes mellitus characterized by insulin resistance and abnormal function of pancreatic beta cells. In recent years, genomic association studies have revealed risk and susceptibility genes associated with genetic susceptibility to GDM. However, genetic predisposition cannot explain the rising global incidence of GDM, which may be related to the increased influence of environmental factors, especially the gut microbiome. Studies have shown that gut microbiota is closely related to the occurrence and development of GDM. This paper reviews the relationship between gut microbiota and the pathological mechanism of GDM, in order to better understand the role of gut microbiota in GDM, and to provide a theoretical basis for clinical application of gut microbiota in the treatment of related diseases.

**Methods:**

The current research results on the interaction between GDM and gut microbiota were collected and analyzed through literature review. Keywords such as "GDM", "gut microbiota" and "insulin resistance" were used for literature search, and the methodology, findings and potential impact on the pathophysiology of GDM were systematically evaluated.

**Results:**

It was found that the composition and diversity of gut microbiota were significantly associated with the occurrence and development of GDM. Specifically, the abundance of certain gut bacteria is associated with an increased risk of GDM, while other changes in the microbiome may be associated with improved insulin sensitivity. In addition, alterations in the gut microbiota may affect blood glucose control through a variety of mechanisms, including the production of short-chain fatty acids, activation of inflammatory pathways, and metabolism of the B vitamin group.

**Discussion:**

The results of this paper highlight the importance of gut microbiota in the pathogenesis of GDM. The regulation of the gut microbiota may provide new directions for the treatment of GDM, including improving insulin sensitivity and blood sugar control through the use of probiotics and prebiotics. However, more research is needed to confirm the generality and exact mechanisms of these findings and to explore potential clinical applications of the gut microbiota in the management of gestational diabetes. In addition, future studies should consider the interaction between environmental and genetic factors and how together they affect the risk of GDM.

## 1 Introduction

GDM is defined as diabetes with normal glucose metabolism or potentially impaired glucose tolerance before pregnancy that develops or is diagnosed only during pregnancy (Szmuilowicz et al., 2019). More than 80% of pregnant women with diabetes mellitus have GDM. Currently, the incidence of GDM is reported to be 1% to 14% in countries around the world, and the incidence in China is 1% to 5%, with a significant increasing trend in recent years (Practice Bulletin No, 2018; He et al., 2019). The clinical development of GDM is complex and is a specific physiologic process in which a state of physiologic insulin resistance exists. Patients’ glucose metabolism can be normalized after delivery, but the chance of developing diabetes mellitus in the future is increased; and those with severe disease or poor glycemic control during the onset of the disease may have hypertensive disorders of pregnancy, excessive amniotic fluid, premature rupture of membranes, postpartum hemorrhage, fetal distress, macrosomia, fetal growth restriction, hypoglycemia of newborns and other serious harms; and the mother and child’s near and distant complications are increased, which should be given high priority (Practice Bulletin No, 2018). increase and should be given high priority (Practice Bulletin No, 2018; Szmuilowicz et al., 2019). Gestational diabetes mellitus is a high-risk pregnancy and it can seriously jeopardize the health of the mother and child. Before the introduction of insulin maternal, mortality was 27-30% and fetal perinatal mortality was greater than 40%. The factors currently thought to be associated with the development of GDM are autoimmune genetic factors, insulin resistance, and chronic inflammation (Practice Bulletin No, 2018; Szmuilowicz et al., 2019). In addition, environmental factors are considered to be another important modulator of GDM (Jo et al., 2019), yet the exact etiology of GDM remains unclear.

As one of the environmental factors, the influence of the gut microbiota on the development of disease may be as important as genetic factors. The intestinal microbiota is not only an extremely complex and diverse microbial community, but it is also an important component of the human body and is essential for the maintenance of the body’s dynamic physiological balance. There are billions of bacteria living in the human intestinal tract, which constitute the largest human microecosystem-intestinal flora. The gut microbial community is involved in the digestion of food, synthesis of essential vitamins and amino acids, elimination of pathogens, and removal of toxins (Adak and Khan, 2019), and functions as a “microbial organ” through the production of biologically active metabolites that participate in a range of metabolic pathways in the human body. Compared with genetic factors, intestinal flora can be more easily detected and regulated, and therefore receives more attention as a promising approach to prevention and treatment. It is a cutting-edge topic in the field of medicine and life sciences in today’s world. This study will elucidate the molecular mechanism of intestinal flora involved in the occurrence and development of GDM, clarify the significance of interventions such as flora transplantation, probiotics, and dietary fiber for the prevention and treatment of GDM, and lay the foundation for the development and use of probiotics for the prevention and treatment of GDM.

## 2 Etiology and factors affecting GDM

GDM is a transient form of diabetes mellitus caused by insulin resistance and pancreatic beta-cell dysfunction during pregnancy (Alejandro et al., 2020). The main reason why pregnant women cause diabetes mellitus during pregnancy is because their bodies undergo certain physiological changes during pregnancy. During the first trimester of pregnancy, high levels of the human hormones placental lactogen (HPL) and cortisol reduce maternal glucose levels. Increased levels of serum estrogen and luteinizing hormone stimulate additional insulin production and secretion while increasing insulin sensitivity (Callaway et al., 2019). In mid-gestation, with increasing levels of estrogen, progesterone, and placental hormones, pregnant women have increased insulin resistance, decreased insulin sensitivity, and increased fasting and postprandial glucose levels (Tsakiridis et al., 2021). The inability of pregnant women to compensate for the physiologic hormone-driven increase in insulin resistance along with decreased insulin sensitivity results in hyperglycemia, causing the pregnant body to develop gestational diabetes problems (Wang et al., 2019).

Genetic factors may contribute to insulin resistance, β-cell dysfunction, neurohormonal dysfunction, inflammation, oxidative stress, epigenetic modifications, and alterations in the gut microbiota. They are related to the occurrence of GDM. For example, several GDM risk genes were found to have functions in glucose metabolism, insulin synthesis and secretion, and insulin signaling through family linkage analysis and genome-wide association studies (Xie et al., 2023). In addition, researchers explored the susceptibility genes of GDM through case-control studies and found that genes such as TCF7L2, VDR, and IGF2BP2 serve multiple functions such as β-cell function, insulin secretion, peripheral insulin resistance, glucose metabolism, and oxidative stress, conferring genetic susceptibility to GDM (Wei et al., 2021). A family history of T2DM is an important risk factor for the development of GDM. Mothers with first- or second-degree relatives with diabetes have a higher unfavorable risk of BMI and impaired insulin sensitivity (Monod et al., 2023).

Environmental factors are considered to be another important modulator of GDM. Environmental exposures to perfluoroalkyl substances, phthalates, poly-fluoroalkyl substances, polychlorinated biphenyls, and polybrominated diphenyl ethers can hurt glucose levels in pregnant women, especially those of normal weight (Yu et al., 2021; Yao et al., 2023). In addition, prolonged exposure to nitrogen dioxide(NO 2) and carbon monoxide (CO) before pregnancy was significantly associated with an increased risk of GDM (Hehua et al., 2021). Studies have found that higher plasma manganese levels in early pregnancy may also be a potentially important risk factor for GDM (Li Q. et al., 2022). Decreased monocyte counts during pregnancy have been strongly associated with the development of GDM, the development of macrosomia, and the chronic inflammatory state of GDM (Huang et al., 2022). Seasonal variations are significantly and positively associated with the prevalence of GDM (Khoshhali et al., 2021).

High-quality diets before and during pregnancy reduce the risk of developing gestational diabetes, whereas poorer diet quality increases the risk of developing gestational diabetes (Gao et al., 2023). Vitamin B12 deficiency is associated with an increased risk of developing GDM, and attention needs to be paid to the balance of vitamin B12 and folate (He et al., 2020). Some beneficial and commensal gut microorganisms are negatively associated with the development of GDM, while opportunistic pathogenic members are associated with a higher risk of developing GDM (Cortez et al., 2019). Higher thiamine and riboflavin intake during pregnancy is associated with a lower incidence of GDM (Ge et al., 2023). Studies have shown that a variety of non-genetic regulatory factors (such as chemistry, environment, diet, intestinal microorganisms, and drugs) play a key role in the pathogenesis of GDM. Therefore, correction of insulin resistance through non-genetic factors is essential for the quality of life and prognosis of GDM patients.

A growing number of studies have found that gut microbiota is closely associated with the development of GDM (Kijmanawat et al., 2019; Wang et al., 2020; Chen et al., 2021). For example, Rold et al. (2022) found significant differences in the gut microbiota between GDM and non-GDM women in a systematic review. In a case-control study, Liu et al (Liu et al., 2020) collected feces from 45 patients with GDM and 45 healthy controls during early and mid-pregnancy to explore their intestinal flora profile. By using genome sequencing technologies, they found that women with GDM had reduced intestinal flora abundance, particularly a decrease in *Anaplasma* and *Akkermansia*. There was a significant negative correlation between the number of *Akkermansia* and glucose levels, while the relative number of *Faecalibacterium* was positively correlated with the levels of inflammatory mediators. In addition, the researchers transplanted gut microbes from gestational and non-pregnant diabetic patients into GF mice. The results showed that the mice developed symptoms of hyperglycemia. These results suggest that the pattern of changes in the gut microbiota of GDM patients is related to the pathogenesis of the disease.

## 3 Gut microbiota

In recent years, the gut microbiota has been one of the research hotspots in the field of biomedicine. All microorganisms presented on the mucosal surface of the human gastrointestinal tract are collectively referred to as intestinal microbiota. The intestinal flora of the human body is very large, and it contains 100 trillion microflora, equivalent to ten times that of the human body. The bacterium weighs 1.5 kilograms and contains more than 3.3 million genes, more than 150 times the genetic number of the human body (Pitocco et al., 2020), suggesting that genetic modification could play an important role in our bodies. There are over 3,500 known strains of human gut flora. Currently, nine flora have been identified at the taxonomic phylum level. The main dominant groups are *Firmicutes* and *Bacteroidetes*, which account for about 98% of the flora. They are followed by *Actinobacteria* and *Proteobacteria*, with minimal amounts of *Verrucomicrobia, Spirochaete, Fusobacteria*, and unclassified phyla closer to *Cyanobacteria* (Eckburg et al., 2005). *Firmicutes* of gut microbiota have abundance values of up to 50-60% and include a total of about 200 genera. *Bacteroidetes* is numerically second only to *Firmicutes*, accounting for 10-48% of the total flora, containing about 20 genera, and is the second most dominant group in gut microbiota. *Bacteroidetes* are divided into three main groups: *Prevotellaceae*, and *Porphyromonas* (*Hou et al., 2022b*). *Prevotellaceae* is less abundant in the human gut, the proportion is often less than 1%, and most of them are pathogenic. *Actinobacteriota* is not numerically dominant in the human gut. *Bifidobacterium* is one of the common probiotic bacteria. Gut microbiota plays an important role in human health, including the ability to regulate intestinal mucosal permeability, produce antimicrobial substances, participate in the synthesis of nutrients such as bile acids and fats and drug metabolism, and stimulate the development of the immune system, etc; whereas enterotoxins produced by pathogenic bacteria induce elevated inflammatory factors, which in turn lead to infections and an imbalance of the intestinal flora. Several studies have confirmed that intestinal flora is closely related to the pathogenesis of a variety of metabolic disease.

## 4 Changes in gut microbiota during the development of GDM

### 4.1 Significantly increased intestinal flora species in pregnant women with GDM

Numerous longitudinal and cross-sectional human case-control studies as well as animal experiments have revealed changes in the gut microbiota of patients with GDM. *Bacteroidetes* were significantly increased in GDM patients, with an increased abundance of *Bacteroides, and Citrobacter Desulfovibrio* (Su et al., 2021). Sun and colleagues conducted a longitudinal case-control study to explore the dynamics of gut microbiota during pregnancy and its relationship to glucose metabolism during pregnancy. They found that *Bacteroides massiliensis* was associated with GDM status, and *Mycobacterium and Anaerostipes hadrons* were associated with impaired glucose tolerance (Sun et al., 2023). Similar results were obtained in animal experiments. Liu et al. investigated the causal effect of gut microbiota from GDM patients on glucose metabolism in germ-free (GF) mice. They implanted stool samples from donors with gestational diabetes and non-gestational diabetes into GF mice. The results showed that the content of *Bacteroidetes* increased significantly in patients with gestational diabetes (Liu et al., 2019). In addition to *Bacteroidetes*, Fugmann et al. (2015) found an increased proportion of *Prevotella* in GDM. In addition, a study on pregnant women with GDM in Japan found an increased abundance of *Romboutsia*. *Romboutsia* plays an important role in insulin resistance disorders associated with pregnancy (Cortez et al., 2019).

### 4.2 Gut microbiota species significantly reduced in pregnant women with GDM

Pregnant women with GDM had a decreased abundance of Romboutsia, Firmicutes, Actinobacteria, Verrucomicrobia, Ruminococcaceae, Ackermannia, Escherichia-Shigella, Bifidobacterium, Clostridia, rothia, and Corynebacterium (Hu et al., 2021; Zhang et al., 2021). This finding has been confirmed in several human trials. For example, Wang et al. (2020) observed reduced levels of Enterobacteria and Rumenococcaceae in GDM compared to healthy participants. Hu et al. (2021) found a significant decrease in Croatia, Actinobacteria, and Bifidobacterium in GDM patients. Furthermore, in a study by Su et al., the degree of decrease in the abundance of Clostridia, Corynebacterium, and this was shown to be positively correlated with fasting blood glucose, and blood glucose levels at 1 hour and 2 hours postprandial. And the abundance of Ackermannia was also shown to be negatively correlated with 1 h blood glucose and positively correlated with insulin sensitivity (Su et al., 2021). In particular, the abundance of Ackermannia is susceptible to dietary influences, and an increase in the intake of foods rich in crude dietary fiber in the patient’s diet is associated with a significant increase in the abundance of Ackermannia in the intestinal flora (Tanaka et al., 2022) (**Table 1**).

**Table 1:** Gut microbiota and related metabolic changes during GDM.

Author, year	Study Design	Group Setting	Time	Measurements	GDM associated gut microbiota
Wang, 2020(Wang et al., 2020)	Case-control study	59 GDM and 48 non-GDM	24-28 weeks of gestation	16S rRNA gene sequencing	Enterobacteria and Rumenococcaceae were reduced in GDM women
Su, 2021(Su et al., 2021)	Case-control study	21 GDM and 32 non-GDM	24-28 weeks of gestation	16S rRNA gene sequencing	the phylum Bacteroidetes increased in GDM and increased Bacteroides, Incertae, Sedis, Citrobacter, Parabacteroides, and Fusicatenibacter genus.
Sun, 2023(Sun et al., 2023)	Prospective cohort study	120 GDM and 120 non-GDM	during three trimesters	16S rRNA gene sequencing	Bacteroides massiliensis, Mycobacterium and Anaerostipes hadrons were increased in GDM women
Liu, 2020(Liu et al., 2020)	Case-control study	45 GDM and 45 non-GDM	24-28 weeks of gestation	16S rRNA gene sequencing	women with GDM had reduced intestinal flora abundance, particularly a decrease in Anaplasma and Akkermansia
Fugmann, 2015(Fugmann et al., 2015)	Prospective cohort study	42 GDM and 35 non-GDM	3 to 16 months after delivery	16S rRNA gene sequencing	Bacteroidetes and Prevotella were increased in GDM women
Cortez, 2019(Cortez et al., 2019)	cross-sectional study	26 GDM and 42 non-GDM	28-36 weeks of gestation	16S rRNA gene sequencing	Romboutsia was increased in GDM women
Hu, 2021(Hu et al., 2021)	Case-control study	201 GDM and 201 non-GDM	6-15 weeks of gestation	16S rRNA gene sequencing	Croatia, Actinobacteria and Bifidobacterium were reduced in GDM women
Zhang, 2021(Zhang et al., 2021)	Prospective cohort study	128 GDM and 709 non-GDM	22-24 weeks of gestation	16S rRNA gene sequencing	Woman who later progressed to GDM showed decreased Enterobacteria and Rumenococcaceae
Tanaka, 2022(Tanaka et al., 2022)	Case-control study	20 GDM and 16 non-GDM	35-37 weeks of gestation	16S rRNA gene sequencing	Ackermannia was increased in GDM women
Huang, 2023(Huang et al., 2023)	Case-control study	21 GDM and 42 non-GDM	12-28 weeks of gestation	16S rRNA gene sequencing	Woman who progressed to GDM showed increased butyrate

## 5 The role of gut microbiota in the pathogenesis of gestational diabetes mellitus

GM maintains a constant dynamic and homeostatic state. However, at the same time, it can be affected by a variety of factors: diet, antibiotic use, medications, and even the pH of drinking water. Consequences of a high-fat diet include an imbalance of intestinal flora, intestinal dysfunction, increased intestinal permeability, and the escape of toxic substances into the bloodstream, which in turn induces diabetes (Malesza et al., 2021; Ye et al., 2022). In diabetic mice, broad-spectrum antibiotic use exacerbates glucose tolerance and increases insulin secretion. The use of antibiotics further alters the microbial community by decreasing the number of Firmicutes, which in turn leads to disturbed glucose metabolism (Han et al., 2019). It has been found that widespread antibiotic use may promote autoimmunity through gut dysbiosis (Vangoitsenhoven and Cresci, 2020). Yang et al. (2023) induced pancreatic inflammation, β-cell destruction, and insulin-dependent diabetes mellitus in antibiotic-treated wild-type mice, and the results suggest that chemically enriched pathogenic bacteria in gut dysbiosis is sufficient to induce insulin-dependent diabetes after pancreatic translocation. Proton pump inhibitors (PPI) are mainly used to inhibit gastric acid production and to treat peptic ulcers. Treatment with PPI reduces gut microbial diversity (Weersma et al., 2020). The pH of drinking water also affects the composition and diversity of gut bacteria. In summary, multiple factors can influence the gut microbiota. Dysbiosis of gut microbiota is strongly associated with the development of gestational diabetes (Koren et al., 2012; Lin and Zhang, 2017; Fenneman et al., 2020; Doroszkiewicz et al., 2021). Studies have shown that in GDM, intestinal flora participates in insulin resistance, induces chronic inflammation, and affects energy balance and blood glucose metabolism (Lau et al., 2021; Ye et al., 2022; Liu et al., 2023; Wu et al., 2023). This paper reviews the above mechanisms to provide new ideas for the occurrence of gestational diabetes mellitus.

Generally, GDM is a chronic metabolic disease characterized by impaired β-cell function and insulin resistance (Alejandro et al., 2020). Many studies have shown that insulin resistance is closely related to a chronic inflammatory response (Yang et al., 2021). Disturbances in the structure of gut microbiota cause an increase in the number of pathogenic bacteria, resulting in an increase in lipopolysaccharides (LPS) produced by gram-negative bacteria and the activation of toll-like receptor 4 (TLR4) and its downstream factors, which increases intestinal permeability and the amount of endotoxin entering the circulation, as well as the up-regulation of adipose tissue pro-inflammatory cytokine and chemokine expression, which causes the onset of chronic inflammation (Cani et al., 2007). High-fat diets are associated with elevated circulating LPS levels. Liu et al. (2023) found that after 8 weeks of high-fat diet feeding, high-fat diet mice had altered gut microbiota, impaired intestinal barrier function, increased endotoxin release into the bloodstream, increased expression of hepatic inflammatory factors (TNF-α, IL-1β, and IL-6), and exacerbated insulin resistance. Huang et al. (2023) found in animal experiments that highly fermentable dietary fiber (HFDF) increased the abundance of butyrate, reduced placenta-derived inflammation by enhancing the intestinal barrier and inhibiting the transfer of bacterial-derived LPS, and ultimately resisted high-fat diet-induced insulin resistance, suggesting a role for LPS signaling in the development of GDM.

**Figure 1:**
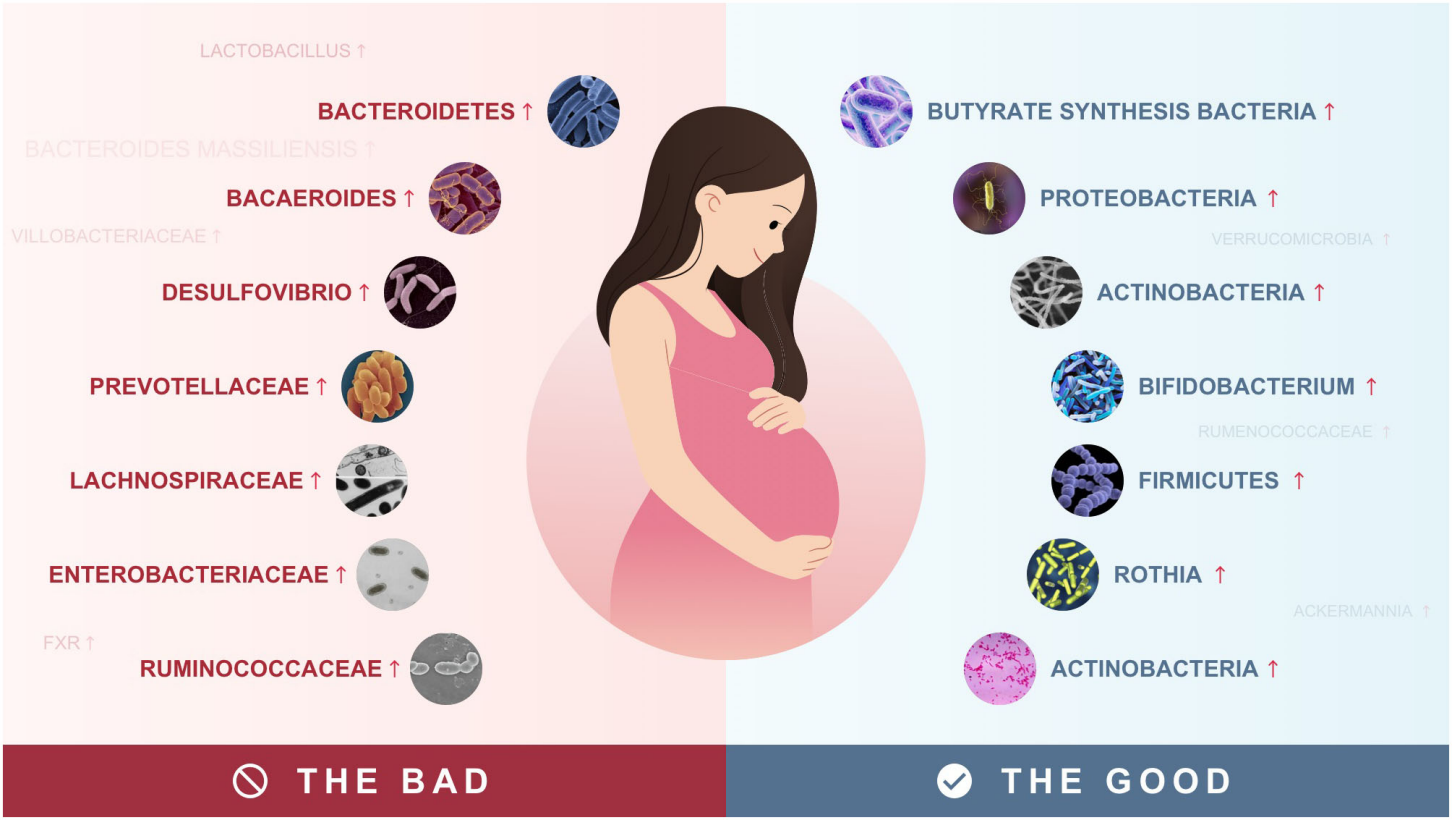
Beneficial and harmful bacteria in the gut during pregnancy.

Studies have shown that intestinal flora has an important effect on the normal physiological function of the body (**Figure 1**). Short-chain fatty acids (SCFAs) including acetic, propionic, and butyric acids are produced by gut microbiota fermenting oligosaccharides, polysaccharides, peptides, proteins, and glycoproteins. These SCFAs have a variety of beneficial effects on energy metabolism in mammals (Topping and Clifton, 2001; Zheng et al., 2020). Recent studies have found that SCFAs are strongly associated with GDM and that abnormal gut microbiota in patients with GDM leads to abnormal SCFAs production (Cortez et al., 2019; Dualib et al., 2021). SCFAs can regulate intestinal mucosal microecology, control the growth of harmful bacteria, maintain the balance between water and electrolytes prevent intestinal mucosal damage, and so on. In addition, SCFAs can also reduce intestinal inflammatory response by inhibiting the secretion of inflammatory cells and promoting the recovery of intestinal inflammatory injury (Ziętek et al., 2021). In the intestines, SCFAs increase the secretion of glucagon-like peptide-1 (GLP-1) mainly by stimulating the signaling pathway of G-protein coupled receptor 41 (GPR41) and GPR43 to achieve the effects of appetite suppression, regulation of intestinal peristalsis and thereby affecting the metabolic absorption of electrolytes and nutrients (Chang et al., 2014; He et al., 2020; Canfora et al., 2022; Liu et al., 2023). Studies have shown that the disorder of intestinal flora can cause the decrease of SCFA production (Qin et al., 2012) and the activity of SCFA receptors, and then cause the disorder of glucose and lipid metabolism and induce GDM (Mokkala et al., 2017).

In addition to SCFAs, we also found that intestinal flora can also participate in the occurrence and development of GDM by regulating cholic acid. Primary bile acids are synthesized in the liver and then circulated to the gut where they are broken down into secondary bile acids by gut microbiota (Liu et al., 2016). Secondary cholic acid can bind to the G protein-coupled receptor TGR5 on the surface of intestinal secretory 1-cells, thus increasing the synthesis of glucagon-like peptide-1 (GLP1) and enhancing insulin sensitivity (Sayin et al., 2013). A study found that bile acid can activate 5-hydroxytryptamine in intestinal chromaffin cells under the condition of intestinal flora disorder, thus reducing the release of insulin and promoting the production of high glucagon (Martin et al., 2019). Mouse experiments show that differences in bacterial composition and metabolism in the gut and bile modulate intestinal Farnesoid X receptor (FXR) signaling, and that elevated concentrations of FXR excitatory factors increase the incidence of metabolic diseases (Sun et al., 2021). Eight metabolites associated with GDM include bile acids, taurocholic acid, glycocholic acid, glycochenodeoxycholic acid, deoxycholic acid, lithocholic acid, ursodeoxycholic acid and taurocholic acid (Wu et al., 2023). Among them, multivariate analysis showed that TCA and LCA were linearly positively and negatively correlated with the risk of GDM respectively. In addition, elevated serum total bile acid concentration was positively associated with the risk of GDM and increased the risk of adverse pregnancy outcomes such as delivery of macrosomic babies and premature rupture of membranes. Therefore, high levels of total bile acid are also considered a risk factor for GDM (Maghsoodi et al., 2019).

Branched-chain amino acids (BCAAs) are hydrolyzed by proteolytic enzymes produced by the intestinal microflora. They contain mainly leucine, valine, and isoleucine. It has been found that diabetic patients have significantly higher serum levels of BCAAs compared to the healthy population (Sun et al., 2021). Phosphorylation of protein kinase B (Ser473 and Ser474) was found in mice fed with BCAAs, which can block normal insulin signaling and cause insulin resistance (Zhang et al., 2022). White et al. (2021) found that lowering plasma BCAAs levels by drugs can improve insulin resistance. The effects of diet, medications, and other factors on gut flora can lead to changes in the levels of BCAAs, which in turn have a regulatory effect on blood glucose and lead to insulin resistance. A small cohort study of Chinese women found that elevated levels of isoleucine in early pregnancy were significantly associated with the development of subsequent GDM (Jiang et al., 2020). Li et al (Li N. et al., 2022). also demonstrated that BCAAs in pregnancy are strongly associated with the pathogenesis of GDM and that increases in leucine and isoleucine can lead to GDM.

## 6 Intervention of gut microbiota aids in the treatment of GDM

Regulation of intestinal flora is an effective way to prevent and treat diseases caused by the imbalance of intestinal flora. The structural changes of intestinal flora in patients with gestational diabetes mellitus (GDM) are an important basis for the treatment of GDM. Approaches to modulating the balance of gut microbiota include the use of FMT, probiotics, and prebiotics. Here, we will briefly review the advances and challenges of improving gestational diabetes by regulating intestinal flora.

Fecal bacteria transplantation (FMT) refers to the prevention and treatment of parenteral diseases by implanting beneficial bacteria such as probiotics in healthy people into the intestines of patients to reshape new flora (Mahmoudi and Hossainpour, 2023). FMT therapy for CDI has promising clinical applications (Khoruts et al., 2010). Inspired by FMT therapy, the researchers explored the effect of FMT on diabetes (Hanssen et al., 2021; Ng et al., 2022; Zhang et al., 2022). In a study, a high-fat diet combined with streptozotocin (100 mg/kg) was used to construct an animal model of type 2 diabetes, and FMT was used to repair the intestinal microecology. The results showed that FMT significantly reduced the insulin sensitivity of pancreatic islets, attenuated apoptosis of pancreatic islet β-cells, and increased the colonization of beneficial microorganisms in the intestinal tract (Wang et al., 2020). In a non-blind, single-arm intervention trial of FMT involving 17 patients with type 2 diabetes, 20 healthy people served as a control group. The study showed that the intestinal flora of type 2 diabetic patients was altered after the FMT intervention and correlated with an increase in intestinal mucus *Rikenellaceae* and *Anaerotruncus* (Ding et al., 2022). FMT can inhibit the progression of diabetes in several ways, thus delaying the onset of GDM. However, due to the lack of studies in GDM, the efficacy and safety of FMT in GDM are still unclear.

Probiotics are active microorganisms that are beneficial to the host, and they not only promote the growth of beneficial bacteria but also inhibit harmful bacteria (Yoshimoto et al., 2013). Many studies have shown that probiotics can be an effective means of regulating gut microbiota, controlling local and systemic inflammation by increasing intestinal permeability and modulating the secretion of pro-inflammatory mediators, thereby decreasing intestinal permeability and enhancing the immune system, which in turn improves and prevents the onset and progression of GDM (Homayouni et al., 2020). A meta-analysis found that probiotic or commensal microorganism-based nutritional supplements during pregnancy can increase levels of glycolipid metabolism in GDM, suppress inflammatory responses, and reduce high cholesterol levels in infants (Zhou et al., 2021). Probiotic supplements during pregnancy have an impact and effect on weight gain during pregnancy and the prevention of GDM. Some studies have been done in clinical trials. Kijmanawat et al. (2019) treated GDM patients with probiotics supplemented with *Bifidobacterium* and *Lactobacillus*, or a placebo during 24-28 weeks of gestation, and found a decrease in fasting glycemia and an increase in insulin sensitivity with the addition of probiotics to the gut.

However, Callaway et al (Diabetes mellitus in overweight and obese women: findings from the SPRING double-blind randomized controlled trial, 2019). found that probiotics taken in the middle of pregnancy in overweight and obese women did not prevent GDM after 28 weeks of gestation. Meanwhile, in a parallel double-blind, randomized, and placebo-controlled clinical trial, Shahriari et al. (2021) concluded that probiotic supplementation of pregnant women did not seem to reduce the risk of GDM or improve another neonatal and maternal prognosis. In addition to this, the study by Pellonperä et al. (2021) also found that interventions with probiotics during pregnancy appeared to be both safe and well-tolerated, but did not have any benefit in reducing the risk of GDM or improving glucose metabolism in overweight women. It was concluded that differences existed due to factors such as probiotic type, dosage, and timing of addition (Hou et al., 2022a). Therefore, more research is needed in the future to better control the dosage and timing of intestinal flora for the management of gestational diabetes. A series of randomized controlled trials on probiotics for the prevention of GDM are continuously being studied (Davidson et al., 2021) and will also provide more data regarding probiotics for the prevention of GDM.

As the largest exogenous determinant of gut microbiota, dietary patterns, and structure can be used as a therapeutic pathway to re-establish healthy microbiota. Studies have demonstrated that consuming foods higher in dietary fiber reduces the risk of inflammation and mortality, especially in diabetic patients. Dietary fiber helps to remodel the gut microbial ecology, ameliorate ecological dysbiosis, and promote the expansion of SCFAs-producing Prevotella and Bifidobacterium bacteria, which in turn increase fecal and systemic SCFAs concentrations and improve glucose homeostasis (Li et al., 2020; Blaak et al., 2020). Large prospective cohort studies have consistently shown that high dietary fiber intake (25 g/day for women and 38 g/day for men) is associated with a 20-30% lower risk of developing T2DM after correcting for confounders (Weickert and Pfeiffer, 2018). Dietary fiber fermentation contributes to the effect of gut microbiota on glucose regulation during pregnancy (Weersma et al., 2020). A meta-analysis showed that dietary fiber supplementation significantly improved glucolipid metabolism and pregnancy outcomes in patients with GDM. Dietary fiber can be used as adjunctive therapy for GDM, and additional insoluble dietary fiber supplementation is recommended for those patients with poor fasting glucose (Sun et al., 2022).

## 7 Discussion

**Figure 2:**
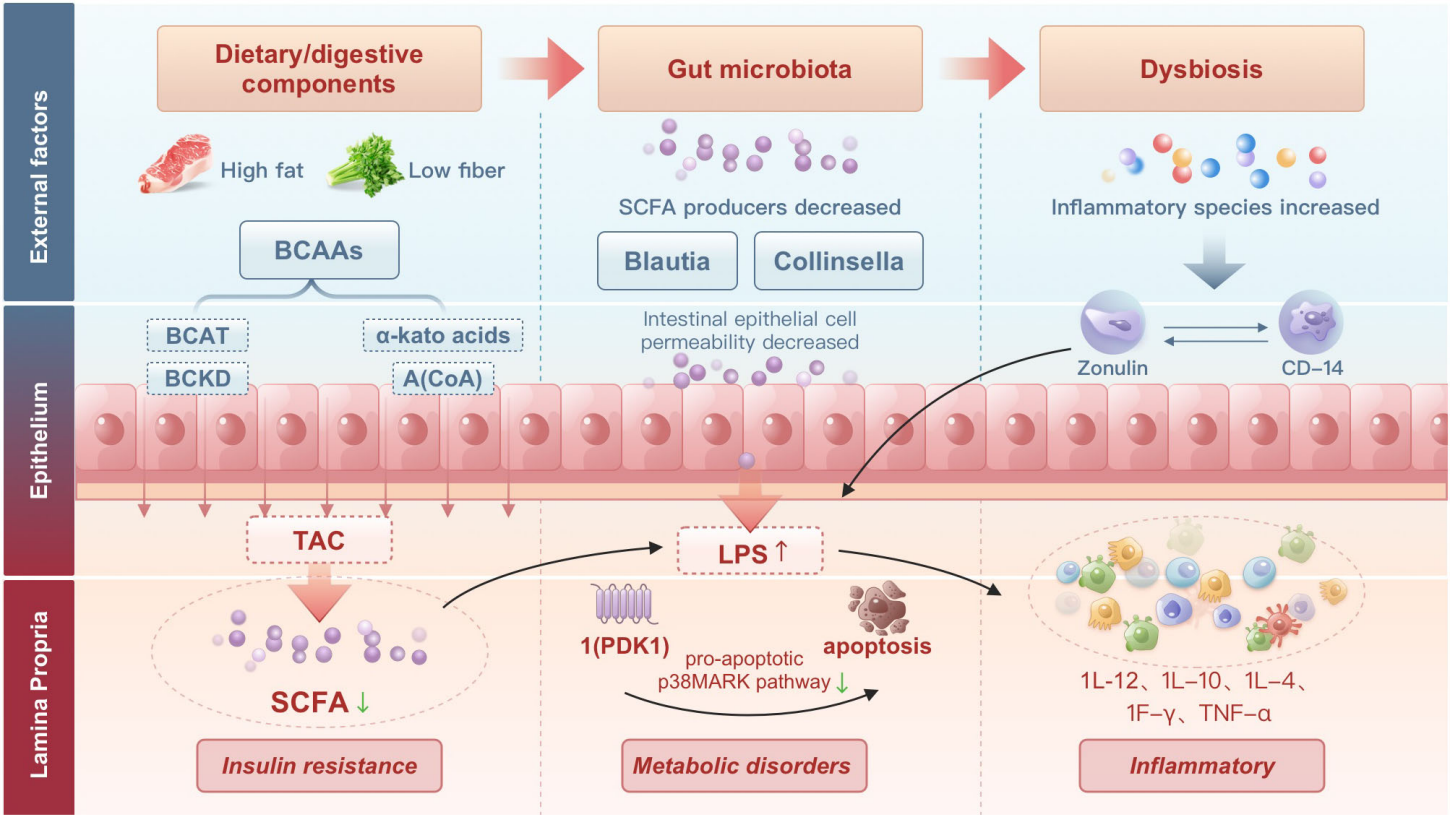
Effects of dietary components on gut microbiota, lipid metabolism, and insulin sensitivity: a visual model of the pathogenesis of gestational diabetes mellitus.

The large number of bacteria in the human intestinal tract constitutes an extremely complex microecological system, which is of great significance to the normal physiological function of the body (Hou et al., 2022a). Recent studies have shown that gut microbiota is closely associated with the onset of GDM (Song et al., 2022). Studies have shown that intestinal flora has multiple regulatory effects on GDM. Here, we review the current evidence that the gut microbiota and the metabolites it produces may drive insulin resistance in GDM by initiating an inflammatory response. Its mechanisms of action are described below (**Figure 2**).

First of all, in GDM, gut microbes may play a role by modulating LPS-induced inflammatory responses. Chronic inflammation is a key feature of GDM. Various inflammatory factors are involved in the development of GDM (Pinto et al., 2023). Disturbed gut microbiota produces large amounts of LPS, which leads to a variety of different biological activities. For example, Liang et al. (2023) found that oral administration of probiotics significantly reduced Gram-negative bacterial counts, lowered inflammatory factor levels, and prevented GDM. Prebiotics such as isomaltodextrin have beneficial effects on chronic inflammation-associated insulin resistance by restoring the intestinal barrier and reducing circulating endotoxin levels (Hann et al., 2019). Unhealthy diet promotes the growth of lipopolysaccharide-producing bacteria such as Enterobacteriaceae, leading to the translocation of LPS through the compromised intestinal barrier, which in turn induces dyslipidemia, insulin resistance, systemic inflammation and immune responses (Sáez-Lara et al., 2016; Ferrarese et al., 2018).

Secondly, the intestinal flora may affect GDM by regulating the flora. Recent studies have shown that microbial metabolites are key factors in the regulation of intracellular glucose metabolism. SCFAs are associated with a number of metabolic processes, including induction of appetite regulation (Byrne et al., 2015; Chambers et al., 2015) and amelioration of insulin resistance in muscle and adipose tissue (Gao et al., 2009; Canfora et al., 2015; den Besten et al., 2015). For example, butyrate inhibits the epigenetic regulator histone deacetylases (HDACs), thereby inducing an anti-inflammatory response, particularly in enterocytes (Lin et al., 2015; Freedman et al., 2018). Propionate and butyrate significantly reduce the inflammation-inducing expression of pro-inflammatory mediators in the placental and adipose tissue of pregnant women. Propionate and butyrate also significantly restored inflammation-induced impaired insulin signaling pathways and insulin-mediated glucose uptake in skeletal muscle in pregnant women (Maghsoodi et al., 2019). High dietary fiber has been reported to reverse insulin resistance, high fasting, and postprandial glucose through microbial fermentation and subsequent production of SCFA, thereby improving glucose and lipid parameters in individuals with diseases associated with metabolic dysfunction (Cronin et al., 2021). SCFAs are key molecules in the regulation of intestinal flora and play an important role in maintaining acid balance, protecting the structure of intestinal epithelial cells, and maintaining the normal physiological function of the body. Thus, SCFAs have become an important target for the prevention and treatment of GDM.

Another major mechanism is bile acid metabolism. Bile acids not only promote lipid transport and intestinal absorption, but also act as inflammatory factors and signaling molecules that can regulate signaling pathways controlling a broad and complex network of costimulatory metabolism, including glucose, lipid, steroid, xenobiotic metabolism as well as regulating energy, through the activation of different bile acid receptors, such as farnesoid X receptor (FXR) and transmembrane G-protein-coupled receptor 5, and the regulation of energy homeostasis, thereby profoundly affecting the metabolic and immune functions (Li and Chiang, 2014; Kiriyama and Nochi, 2019). For example, galacto-oligosaccharides can inhibit the progression of obesity and insulin resistance in mice by increasing the expression of intestinal glucagon-like peptide 1 (GLP1) and decreasing fecal bile acid excretion (Mistry et al., 2020). Long-chain polyphosphate from Lactobacillus brevis improves intestinal inflammation and intestinal barrier function through activation of the extracellular regulatory protein kinase (ERK) signaling pathway (Isozaki et al., 2021). Cholic acid is a new approach with the function of regulating glucose metabolism, which has been widely used in clinics. Therefore, maintaining a balanced intestinal flora is crucial for balancing bile acid metabolism, which is essential for improving GDM.

Finally, gut microbiota can influence GDM by modulating branched-chain amino acid metabolism. BCAAs are important nutrient metabolism signaling scores in the body. Many studies have shown that alterations in the gut microbiota can regulate the metabolism of BCAAs, thereby promoting the development of diabetes. For example, Pedersen et al. (2016) found that *Prevotellaceae* and *Bacaeroides* were the main species driving the association between BCAAs biosynthesis and insulin resistance. In mouse experiments, they demonstrated that *Prevotellaceae* induced insulin resistance, exacerbated glucose intolerance, and increased circulating levels of BCAAs.

## 8 Conclusion and future perspectives

Intestinal flora is considered to be an important regulator of GDM susceptibility and plays an important role in patients with gestational diabetes mellitus, both compositionally and functionally. In patients with GDM, an increase in the number of Bacteroidetes, as well as a decrease in Firmicutes, Proteobacteria, and Actinobacteria are common, which may be the main cause of GDM. Several factors associated with gut flora in GDM have been elucidated, including LPS, SCFAs, Bile acids, and BCAAs. The intestinal flora may not only be used as a diagnostic biomarker, but also a potential therapeutic target for GDM. However, the exact driver bacteria and flora are unknown. Therefore, multicenter studies are needed. In addition, multi-omics has been widely used in gut microbiology studies, such as metagenomics and metabolomics, to explore the role of gut flora in GDM. Elucidating the exact role and mechanisms of gut flora in GDM will provide new insights for developing individualized treatments for patients with GDM.

## Data availability statement

The original contributions presented in the study are included in the article/supplementary material. Further inquiries can be directed to the corresponding authors.

## Author contributions

SM: Formal analysis, Methodology, Resources, Writing – original draft. YW: Methodology, Writing – original draft, Conceptualization, Investigation. XJ: Methodology, Writing – original draft, Resources. SD: Methodology, Writing – original draft. SW: Writing – original draft, Resources, Software. SZ: Writing – original draft, Data curation, Formal analysis. FD: Data curation, Writing – original draft, Resources. JC: Writing – original draft, Methodology, Software. BL: Software, Writing – original draft, Resources. BK: Software, Writing – original draft, Data curation. WL: Funding acquisition, Supervision, Writing – review & editing. KH: Writing – review & editing, Resources, Supervision.
